# Pivotal Role of Corneal Fibroblasts in Progression to Corneal Ulcer in Bacterial Keratitis

**DOI:** 10.3390/ijms22168979

**Published:** 2021-08-20

**Authors:** Teruo Nishida, Koji Sugioka, Ken Fukuda, Junko Murakami

**Affiliations:** 1 Department of Ophthalmology, Yamaguchi University Graduate School of Medicine, Ube, Yamaguchi 755-8505, Japan; tnishida@cg8.so-net.ne.jp; 2 Division of Cornea and Ocular Surface, Ohshima Eye Hospital, Fukuoka 812-0036, Japan; 3 Department of Ophthalmology, Kindai University Nara Hospital, Ikoma, Nara 630-0293, Japan; sugioka@med.kindai.ac.jp; 4 Department of Ophthalmology and Visual Science, Kochi Medical School, Kochi University, Nankoku, Kochi 783-8505, Japan; 5 Division of Ophthalmology, Sakibana Hospital, Izumi, Osaka 594-1105, Japan; ophthalmosaurus.discus@gmail.com

**Keywords:** corneal fibroblast, corneal ulcer, bacterial keratitis, collagen degradation, *Pseudomonas aeruginosa*, *Staphylococcus aureus*, matrix metalloproteinase, polymorphonuclear neutrophil, cytokine

## Abstract

The shape and transparency of the cornea are essential for clear vision. However, its location at the ocular surface renders the cornea vulnerable to pathogenic microorganisms in the external environment. *Pseudomonas aeruginosa* and *Staphylococcus aureus* are two such microorganisms and are responsible for most cases of bacterial keratitis. The development of antimicrobial agents has allowed the successful treatment of bacterial keratitis if the infection is diagnosed promptly. However, no effective medical treatment is available after progression to corneal ulcer, which is characterized by excessive degradation of collagen in the corneal stroma and can lead to corneal perforation and corneal blindness. This collagen degradation is mediated by both infecting bacteria and corneal fibroblasts themselves, with a urokinase-type plasminogen activator (uPA)-plasmin-matrix metalloproteinase (MMP) cascade playing a central role in collagen destruction by the host cells. Bacterial factors stimulate the production by corneal fibroblasts of both uPA and pro-MMPs, released uPA mediates the conversion of plasminogen in the extracellular environment to plasmin, and plasmin mediates the conversion of secreted pro-MMPs to the active form of these enzymes, which then degrade stromal collagen. Bacterial factors also stimulate expression by corneal fibroblasts of the chemokine interleukin-8 and the adhesion molecule ICAM-1, both of which contribute to recruitment and activation of polymorphonuclear neutrophils, and these cells then further stimulate corneal fibroblasts via the secretion of interleukin-1. At this stage of the disease, bacteria are no longer necessary for collagen degradation. In this review, we discuss the pivotal role of corneal fibroblasts in corneal ulcer associated with infection by *P. aeruginosa* or *S. aureus* as well as the development of potential new modes of treatment for this condition.

## 1. Introduction

The transparency of the cornea allows light to enter the eyeball. In addition, the smooth surface and convex curve of the cornea support the focusing of images of the outside world on the retina. The loss of these properties of the cornea therefore impairs visual acuity and diminishes quality of life. As a consequence of its location at the ocular surface, the cornea is exposed to pathogens such as bacteria in the external environment. However, infection of the cornea occurs only rarely as a result of the operation of various protective mechanisms. Physically, the corneal surface is protected by the eyelids and is covered by tear fluid [1]. Tear fluid serves not only as a lubricant and wetting solution for the ocular surface, however, but also as a source of biological protective factors including components of the innate immune system. Indeed, it contains more than 60 proteins [2], some of which are bactericidal, with important constituents including inflammatory cytokines, lactoferrin, lysozyme, immunoglobulin A, and members of the cationic antimicrobial peptide family [3,4,5,6,7,8,9,10]. In addition to these biochemical components, the mechanical drainage and turnover of tear fluid promoted by blinking serve to maintain the ocular surface in a clean condition. As an additional and important means of physical protection, the most superficial layer of the corneal epithelium provides a robust barrier to external agents that is dependent on the formation of tight junctions between neighboring cells [11,12,13,14,15,16].

Infectious keratitis caused by bacteria, fungi, protozoa, or viruses is a leading cause of corneal blindness, despite the development of various antimicrobial drugs [17,18]. Bacterial keratitis accounts for most cases of infectious keratitis [19,20,21,22,23,24]. In developing countries, trauma to the ocular surface is the most common risk factor for bacterial keratitis. On the other hand, the wearing or inappropriate handling of contact lenses is the predominant risk factor in developed countries [24,25,26,27]. Ocular surface diseases such as dry eye and neurotrophic keratopathy, corneal surgery, and systemic conditions such as diabetes mellitus can also predispose individuals to the development of bacterial keratitis. The Gram-negative bacterium *Pseudomonas aeruginosa* and the Gram-positive bacterium *Staphylococcus aureus* are the two most frequent causative agents of infectious keratitis [28].

Despite the recent emergence of drug-resistant strains of bacteria, which is a serious clinical problem, the administration of antimicrobial agents is the mainstay of treatment for bacterial keratitis at its early stages. The development of new drugs with different mechanisms of action is an urgent need; however, in order to avoid the increasing problem of drug resistance. With regard to the advanced stage of bacterial keratitis, which manifests as corneal ulcer and subsequent corneal perforation, there is currently no treatment modality available to arrest the progression of corneal stromal melting. Although the excessive degradation of stromal collagen that gives rise to corneal ulcer has been found to be mediated by a urokinase-type plasminogen activator (uPA)-plasmin-matrix metalloproteinase (MMP) cascade, no treatment targeted to this cascade has yet been established.

Excellent reviews on the epidemiology, bacteriology, and clinical treatment of infectious keratitis have been published [19,20,29,30,31,32,33]. In this review article, we address the role of corneal fibroblasts in the metabolism of collagen in the corneal stroma, how bacterial invasion disrupts this homeostatic role, and potential new directions for the development of novel therapies for corneal ulceration [34,35,36]. To help identify the relevant literature for this review, we searched PubMed and Google Scholar with the use of broad terms relating to the etiology, epidemiology, pathobiology, management, and therapy of bacterial keratitis or corneal ulcer.

## 2. Onset and Clinical Course of Bacterial Keratitis

The onset and course of bacterial keratitis can be divided into various phases (Table 1). Events that precede the development of bacterial keratitis include both the adhesion of bacteria in the external environment to the ocular surface and disruption of the corneal epithelial barrier by trauma, contact lens wear, or other insults. In the case of individuals with severe ocular allergic diseases, allergic inflammation in the conjunctiva is associated with the release of various cytokines and cytotoxic mediators—such as interleukin (IL)-4, IL-13, and tumor necrosis factor-α (TNF-α)—from T lymphocytes as well as of cytotoxic proteins from eosinophils [37]. These factors can damage corneal epithelial cells and disrupt the barrier function of the corneal epithelium [13,38,39,40].

The onset of bacterial keratitis (phase 1) begins with the penetration of bacteria into the corneal epithelium and superficial portion of the stroma and their subsequent replication in the host tissue. This penetration of the corneal epithelium can result in the release of alarmins by damaged epithelial cells. At this stage, the infection can be diagnosed on the basis of clinical signs and symptoms, smear culture, and detection of bacterial DNA by the polymerase chain reaction, and it can be halted by the administration of appropriate antimicrobial agents. Prompt diagnosis and treatment can thus lead to the resolution of most cases of bacterial keratitis.

In infectious keratitis, keratocytes of the corneal stroma, rather than corneal epithelial cells, are responsible for innate immune responses. Keratocytes or corneal fibroblasts (activated keratocytes) recognize bacterial components through members of the Toll-like receptor (TLR) family of proteins [41,42,43]. Lipopolysaccharide (LPS), a component of the cell membrane of Gram-negative bacteria, and peptidoglycans derived from the cell membrane of Gram-positive bacteria are recognized primarily by TLR4 and TLR2, respectively, expressed on the surface of stromal fibroblasts. These pathogen-associated molecular patterns (PAMPs) induce the expression of various chemokines and adhesion molecules in corneal fibroblasts [41]. Tear fluid of healthy humans contains high concentrations of LPS binding protein (LBP) and soluble CD14 (sCD14) [44], and these two soluble factors potentiate the LPS-induced innate immune responses of corneal fibroblasts [45]. LPS released from Gram-negative bacteria such as *P. aeruginosa*, together with LBP and sCD14, induces the production of uPA and MMPs in addition to expression of the chemokine IL-8 and the adhesion molecule ICAM-1 (intercellular adhesion molecule-1) by corneal fibroblasts [36,45,46,47]. The expression of IL-8 and ICAM-1 promotes the local infiltration and activation of polymorphonuclear neutrophils (PMNs), and these cells then contribute to clearance of the pathogen [48].

Phase 2 of bacterial keratitis is characterized by the further penetration of actively replicating bacteria into the corneal stroma and the onset of corneal ulceration, which is defined as the destruction of the normal architecture of the corneal stroma associated with the melting of stromal collagen. Bacteria themselves secrete collagen-degrading enzymes that mediate direct collagen degradation by the bacterial cells. However, it is corneal fibroblasts, whose activation is triggered by factors released from the bacteria, that are largely responsible through the production of collagen-degrading MMPs for the destruction of extracellular collagen associated with corneal ulcer. Corneal fibroblasts thus serve as sentinel cells, local immune modulators, and mediators of collagen degradation in infectious keratitis [34,35,36]. MMP1 is the principal mediator of such collagen degradation [35,49,50,51]. It and other soluble MMPs are synthesized and released as proenzymes that undergo proteolytic activation by plasmin or other proteases, with plasmin in turn being produced as a result of the proteolytic conversion of plasminogen by uPA, which is also produced by corneal fibroblasts. The uPA-plasmin-MMP cascade thus plays a central role in the excessive collagen degradation that occurs during corneal ulceration. 

The further infiltration and activation of inflammatory cells in the infected cornea mediated by chemokines, adhesion molecules, and other cytokines contribute to phase 3 of bacterial keratitis. These inflammatory cells—in particular, PMNs—release additional cytokines such as IL-1 that further stimulate corneal fibroblasts. The interaction of PMNs and corneal fibroblasts results in the operation of a vicious cycle [52], with the prolonged and uncontrolled activation of corneal fibroblasts by PMNs giving rise to melting of the corneal stroma. There is currently no medical treatment available to arrest the collagen destruction at this phase. For cases of bacterial keratitis that have progressed to phase 3, the clinical outcome is thus poor, with the resulting corneal ulceration and, in some instances, corneal perforation potentially necessitating corneal transplantation.

With regard to therapeutic strategies for bacterial keratitis, elimination of the causative agent with antimicrobials is thus the first choice for cases at phase 1 or 2. Prompt presentation to an ophthalmologist, proper diagnosis, identification of the causative bacterial species, and administration of treatment are essential to avoid progression to corneal ulcer. No reliable drug treatment is available for cases that have progressed to phase 3, with a poor prognosis characterized by the loss of corneal transparency, and visual acuity being the likely outcome. Regardless of the causative bacteria, the first 24 to 48 h after the onset of bacterial keratitis correspond to phase 1 and early phase 2. However, further progression depends on the causative agent and host factors including predisposing conditions. The duration of phases 2 and 3 thus varies among individuals, with detailed clinical observation being important to identify disease stage.

## 3. Culture Model for Measurement of Collagen Degradation by Corneal Fibroblasts

In the corneal stroma, keratocytes are embedded in a tightly packed extracellular matrix consisting mostly of type I collagen, with cellular functions being continuously modulated by interaction with this matrix. Keratocytes in the healthy cornea manifest a flattened and stellate morphology. They elaborate cytoplasmic processes that are connected to those of neighboring cells by gap junctions, thereby forming a three-dimensional network structure [53,54]. Although keratocytes are dispersedly distributed between the lamellae of stromal collagen fibers, their activities are synchronized by the exchange of intracellular molecules and ions through the gap junctions. The balance between the synthesis and degradation of collagen by keratocytes plays a key role in the maintenance of stromal homeostasis. The morphologic and biochemical characteristics of corneal fibroblasts cultured in a collagen gel differ from those of the cells cultured on a plastic tissue culture dish [55]. An extracellular collagen matrix also promotes IL-1β-induced uPA expression in corneal fibroblasts [56]. Three-dimensional culture of keratocytes (corneal fibroblasts) in a collagen gel has thus been found to recapitulate more closely the in vivo condition compared with monolayer culture [55].

In pathological conditions, keratocytes transform into corneal fibroblasts, and activation of the uPA-plasmin-MMP cascade is central to the associated degradation of stromal collagen. The homeostatic balance of collagen maintenance is thus tilted in favor of collagen degradation with the onset of bacterial keratitis. The components of the uPA-plasmin-MMP cascade as well as upstream regulatory factors have been well investigated at the molecular level in vitro, including analyses of their expression at the mRNA and protein levels and of their activity. Furthermore, insight into the pathobiology of corneal ulceration in phases 2 and 3 of bacterial keratitis has been provided by measurement of collagen degradation in collagen gel cultures. Infiltrated PMNs also come into contact with resident corneal fibroblasts and stromal collagen during bacterial keratitis. The functions of PMNs are also affected by this extracellular matrix, with the release of IL-1 and IL-8 by these cells having been found to be stimulated and inhibited, respectively, by culture in a collagen gel [52,57].

The collagen-degrading activity of corneal fibroblasts cultured in a three-dimensional collagen matrix can be determined directly by measurement of collagen breakdown products [58]. The advantage of this experimental model is that the cells that synthesize and secrete MMPs are embedded in a collagen matrix that is also a substrate of MMPs. The cells are thus embedded in a matrix of type I collagen and cultured in serum-free medium. At various intervals, the culture medium is collected and subjected to ultrafiltration in order to remove intact collagen fibrils with a molecular mass of >100 kDa. The remaining small-sized collagen fragments present in the filtrate are then subjected to hydrolysis by heat and acid treatment, after which the amount of released hydroxyproline is measured with a colorimetric assay. In the presence of plasminogen, the extent of collagen degradation by the corneal fibroblasts depends on both the culture period and the number of cells [58]. The addition of various inflammatory mediators such as cytokines, of sterile culture supernatants of bacteria, or of other cells such as PMNs to the culture system allows the study of interactions among these components under conditions that resemble those in the infected corneal stroma. This in vitro culture model thus revealed, for example, that the addition of the inflammatory cytokine IL-1 stimulates collagen degradation by corneal fibroblasts in a concentration-dependent manner [58] and that both *P. aeruginosa* and *S. aureus* also enhance fibroblast-mediated collagen degradation [59].

## 4. Molecular Mechanisms of Corneal Ulceration

**Figure 1:**
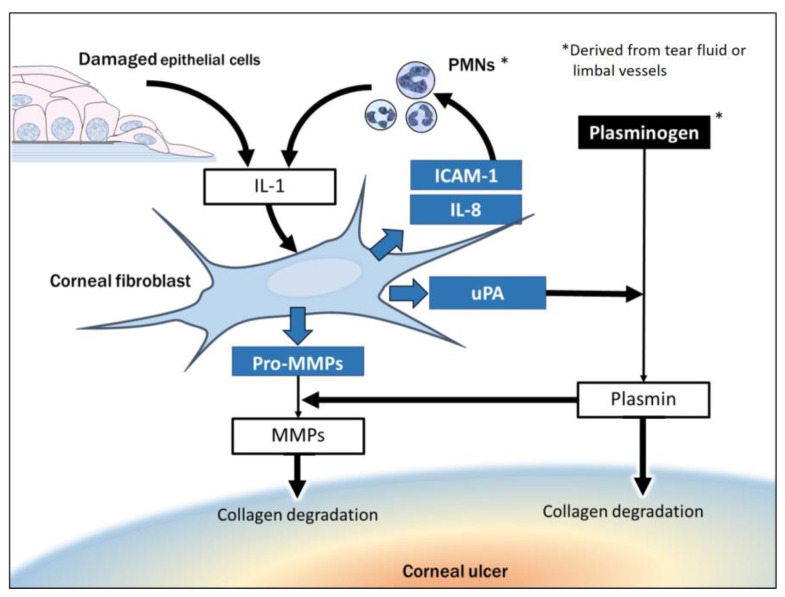
Pathway of collagen degradation mediated by corneal fibroblasts. IL, interleukin; PMN, polymorphonuclear neutrophil; ICAM-1: intercellular adhesion molecule-1; uPA: urokinase-type plasminogen activator; MMP: matrix metalloproteinase.

Uncontrolled degradation of collagen in the corneal stroma underlies the development of corneal ulcer. The degradation of collagen is mediated by cleavage of collagen fibers by extracellular proteases, endocytosis of collagen fragments as a result of their interaction with collagen receptors, and further intracellular proteolysis within lysosomes [60,61]. Two principal pathways of collagen destruction are operative during bacterial keratitis: (1) collagen degradation mediated directly by proteases released by bacteria, and (2) that mediated by corneal fibroblasts (Figure 1). Collagen degradation by corneal fibroblasts is induced by the interaction of these cells with PAMPs of bacteria, with infiltrated PMNs or their secreted cytokines, or with alarmins released by damaged corneal epithelial cells. In the clinical setting, which pathway of collagen destruction is most active in a given patient at a specific time has important ramifications for therapeutic options. Whereas the administration of antimicrobials to eliminate the causative bacteria has the potential to halt collagen degradation by pathway 1, no drug is available to arrest that by pathway 2, although steroids may ameliorate associated inflammation (Table 1). During phase 1 or early phase 2 of bacterial keratitis, only pathway 1 is active, with the result that administration of appropriate antimicrobials is essential to halt progression to late phase 2 and phase 3. However, after corneal fibroblasts are activated and the disease progresses through phase 2 to phase 3, corneal collagen destruction by pathway 2 may proceed regardless of the absence or presence of the causative bacteria.

### 4.1. Direct Collagen Destruction by Factors Released from Bacteria

**Figure 2:**
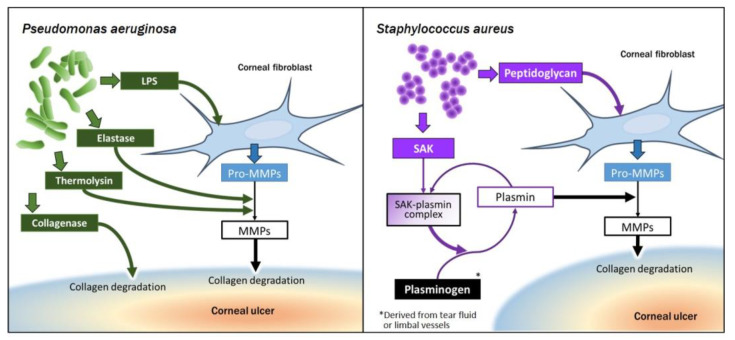
Pathways by which infection with *Pseudomonas aeruginosa* or *Staphylococcus aureus* promotes collagen degradation mediated by corneal fibroblasts. LPS: lipopolysaccharide; MMP: matrix metalloproteinase; SAK: staphylokinase.

The clinical findings of bacterial corneal ulcer depend on the type of infecting bacterium [28,62], in part because of differences in the virulence factors and enzymes released by the bacterial cells [30]. *Pseudomonas aeruginosa* and *S. aureus* are the most common bacteria isolated from infectious corneal ulcer [20]. The former is a Gram-negative bacterium that is ubiquitous in the natural environment, including soil and water, whereas the latter is an anaerobic Gram-positive coccus that is found in the pharynx as well as on the skin, including the perineum and axillae [22]. An understanding of how *P. aeruginosa* and *S. aureus* are able to cause infectious corneal ulcer is essential to devising new treatment strategies (Figure 2).

Proteases capable of degrading collagen are referred to as collagenolytic enzymes and include certain microbial proteases as well as mammalian MMPs and cysteine proteases [63]. Microbial collagenases have been found to be produced by several pathogenic bacteria and fungi [64], with the bacterial enzymes including metalloproteinases and serine proteases [63].

In *P. aeruginosa* keratitis, progression of corneal ulcer is relatively rapid. Elastase and alkaline protease released by *P. aeruginosa* have been shown to contribute to the destruction of corneal collagen and corneal structure [65,66]. The bacteria also release pseudomonal collagenase and other proteinases, with the collagenase being capable of degrading stromal collagen directly and both elastase and thermolysin possessing the ability to convert pro-MMPs to active MMPs.

With the use of the three-dimensional gel culture system, it was shown that sterile culture broth of *P. aeruginosa* degrades collagen to some extent in the absence of corneal fibroblasts, indicating that bacterial virulence factors alone possess collagenolytic activity. However, the culture broth markedly stimulated collagen degradation mediated by embedded corneal fibroblasts [59]. These results thus reflect the intrinsic collagenolytic activity of virulence factors released by *P. aeruginosa* as well as the ability of such factors (including LPS) to stimulate the production of MMPs by corneal fibroblasts and that of factors (including elastase and thermolysin) to activate pro-MMPs.

In the case of *S. aureus*, cysteine proteases known as staphopains are secreted by the bacterial cells and are capable of degrading collagen [67]. However, in contrast to *P. aeruginosa*, sterile culture broth of *S. aureus* manifested direct collagenolytic activity in a three-dimensional collagen gel only in the presence of plasminogen. Another factor released by *S. aureus* is staphylokinase (SAK), which binds to trace amounts of plasmin to form an SAK–plasmin complex that manifests uPA-like activity. The direct collagen-degrading activity of *S. aureus* culture broth thus appears to be actually mediated by plasmin generated from plasminogen by the SAK–plasmin complex. The uPA-like activity of this complex also seems to be responsible in part for the promotion by the sterile culture broth of collagen degradation mediated by corneal fibroblasts [68].

### 4.2. Cell-Dependent Collagen Destruction

Corneal fibroblasts play a central role as both sentinel cells and modulators of local immune reactions during bacterial keratitis [36]. They also directly contribute to the development of infectious corneal ulcer as key effector cells of collagen degradation [35]. In addition to the release of virulence factors that mediate direct collagen degradation, bacteria release factors that stimulate the production of pro-MMPs and uPA by corneal fibroblasts. The released pro-MMPs are processed by plasmin to yield the active form of the enzymes, certain of which are then able to degrade collagen [49]. The contribution of corneal fibroblasts to collagen degradation is thus dependent on the uPA-plasmin-MMP cascade, with the role of uPA being to generate plasmin from plasminogen. The activity of MMPs is also regulated by specific inhibitory factors including tissue inhibitors of matrix metalloproteinases (TIMPs) and α2-macroglobulin [69]. In addition to plasmin, secreted pro-MMPs can be activated by various other proteases including trypsin, chymase, elastase, and certain metalloproteinases [70].

#### 4.2.1. Collagen Degradation Resulting from Exposure of Corneal Fibroblasts to Alarmins

At the onset of bacterial keratitis, infection damages corneal epithelial cells and triggers the infiltration of inflammatory cells including macrophages and PMNs. The damaged epithelial cells release various endogenous molecules known as alarmins that signal danger to surrounding tissue [71]. Alarmins comprise a heterogeneous group of molecules that include IL-1α. Those released from necrotic corneal epithelial cells up-regulate the expression of IL-6 and IL-8 in and attenuate the barrier function of neighboring intact epithelial cells [38]. They also act on corneal fibroblasts to stimulate the expression of MMP1 and down-regulate that of TIMP1 [72]. These actions of alarmins released from necrotic corneal epithelial cells were found to be inhibited by IL-1 receptor antagonist and by antibodies to IL-1α, suggesting that they are mediated by IL-1α.

Both IL-1α and IL-1β bind to the same IL-1 receptor and show identical biological activities [73]. The proinflammatory cytokine IL-1 has been shown to regulate the expression of MMPs in several cell types including dermal fibroblasts [74], cardiac fibroblasts [75], chondrocytes [76], mesenchymal stem cells [77], and corneal fibroblasts [78]. IL-1β was thus found to stimulate the production of MMP1, MMP3, and MMP9 by corneal fibroblasts cultured in a collagen gel as well as to increase collagen degradation by corneal fibroblasts in this culture system [58,79]. The signal transduction mechanism responsible for matrix degradation induced by IL-1β appears to differ depending on target cell type [74,75,80,81]. The transcription factor NF-κB (nuclear factor-κB) contributes to the IL-1β-induced degradation of collagen by corneal fibroblasts [82].

#### 4.2.2. Collagen Degradation Due to Interaction of Corneal Fibroblasts with Infiltrated PMNs

Bacterial infection triggers the recruitment of PMNs to the infection site by activation of an innate immune reaction. The infiltrated PMNs then secrete IL-1, which, as mentioned above, is also released by damaged epithelial cells as an alarmin. IL-1 plays a key role in regulation of the host response to bacterial infection including that associated with corneal ulcer. IL-1 contributes to corneal tissue destruction in part by stimulating the production by corneal fibroblasts of chemokines such as IL-8 that prolong the infiltration of PMNs in the cornea [83]. The mutual interaction of resident corneal fibroblasts with infiltrated neutrophils promotes excessive degradation of stromal collagen. The addition of neutrophils to corneal fibroblasts in the collagen gel culture system was thus found to increase the amount of collagen degraded by the fibroblasts, with this effect being attributed in part to the secretion of IL-1 by the neutrophils [52]. The adhesion molecule ICAM-1 facilitates PMN recruitment, and its expression by corneal fibroblasts is up-regulated after P. aeruginosa infection in vivo, resulting in the ICAM-1-mediated adhesive interaction of corneal fibroblasts with infiltrating PMNs [84,85,86]. The activation of corneal fibroblasts by factors derived from bacteria or damaged epithelial cells is thus followed by the recruitment and activation of inflammatory PMNs mediated by up-regulation of IL-8 and ICAM-1 expression in corneal fibroblasts. The further stimulation of corneal fibroblasts by IL-1 released from PMNs completes a vicious cycle, with the activation of corneal fibroblasts also being accompanied by increased synthesis and secretion of MMPs.

#### 4.2.3. Collagen Degradation Resulting from Interaction of Corneal Fibroblasts with Bacteria

The role of the interaction of corneal fibroblasts with pathogen-derived factors in collagen degradation has been investigated with the use of the collagen gel culture system (Figure 2). *Pseudomonas aeruginosa* releases both bacterial collagenase, which directly degrades collagen [59], as well as elastase and thermolysin, both of which activate pro-MMPs released from resident corneal fibroblasts and thereby indirectly promote collagen degradation in a manner independent of uPA and plasmin [34,59,66]. In addition, LPS derived from the outer membrane of *P. aeruginosa* is recognized by TLR4 expressed at the surface of corneal fibroblasts [87,88] and induces severe corneal inflammation in vivo [89]. Infection with *P. aeruginosa* thus promotes collagen degradation by corneal fibroblasts through pathways both dependent on and independent of the uPA-plasmin system.

In addition to the direct collagenolytic activity of secreted enzymes, *S. aureus* also stimulates collagen degradation by corneal fibroblasts. Sterile culture broth of *S. aureus* thus up-regulates the production of pro-MMPs and uPA by corneal fibroblasts [68], with this effect being due in part to bacterial peptidoglycans. Furthermore, the SAK-plasmin complex promotes the activation of pro-MMPs by mediating the conversion of plasminogen to plasmin. Both of these mechanisms by which *S. aureus* stimulates collagen degradation by corneal fibroblasts are thus dependent on plasminogen.

## 5. Exploration of New Medical Treatment Modalities for Corneal Ulcer

Effective drug treatment for bacterial corneal ulcer remains to be established. The preferred treatment for the initial stages of bacterial keratitis is the administration of appropriate antibiotics. Eyedrops containing broad-spectrum antibiotics are thus often applied, although no such antibiotic is effective against all potential pathogens. One disadvantage of antibiotic therapy is that it can induce the rapid release of LPS from Gram-negative bacteria [90]. Moreover, even if the pathogen is sensitive to the administered antibiotic, collagen degradation and the progression to corneal ulcer can proceed independently of the presence of bacteria after keratocytes have undergone the transition to corneal fibroblasts. Antibiotics are not able to attenuate the inflammatory responses of corneal fibroblasts and infiltrated PMNs. Whereas the recruitment of PMNs to an infection site is a favorable reaction to protect the body from insults, the persistence of the infiltrated cells can result in damage to the host tissue. The development of new therapeutic agents able to reduce inflammation and attenuate the keratocyte activation that results in corneal melting is thus an urgent need for improving the clinical outcome of bacterial keratitis.

MMPs are a potential therapeutic target to prevent collagen degradation by corneal fibroblasts. However, whereas drugs that are able to inhibit MMP activity in vitro have been developed, none to date has shown any clinical benefit with regard to halting the progression of corneal ulcer. Agents including citrate [91], the chelator EDTA, a thiol-containing peptide [92,93], cysteine [94], and a synthetic inhibitor of MMPs [59,79,95] have thus been investigated for their potential as therapeutic agents for corneal ulcer in the clinical setting, but no favorable results have been obtained, even though all of these agents inhibit MMP activity in vitro and showed promising results in animal models. Given that corneal fibroblasts are the source of MMPs responsible for stromal collagen melting, the targeting of these cells rather than MMPs themselves is a potential alternative therapeutic approach for corneal ulcer [34]. 

Doxycycline is a long-acting tetracycline antibiotic but also possesses MMP-inhibitory activity [96,97,98,99]. It has thus been administered as an adjunctive therapy for corneal ulceration associated with bacterial keratitis on the basis of its ability to inhibit collagen destruction and its antibiotic activity [100,101]. It has also been shown to attenuate the infiltration of neutrophils into the cornea [102]. However, no definitive clinical trials of the efficacy of doxycycline for the treatment of corneal ulcer have been reported.

Steroids are currently the main class of drugs administered for amelioration of corneal ulcer (Table 1). If bacteria have been killed and eliminated from the infection site, steroids can be applied to improve outcome by minimizing corneal haze, scarring, and opacity. It is potentially dangerous to administer steroid treatment if bacteria remain at the infection site, however, because of the immunosuppressive action of these drugs. In general, steroids are applied as an adjunctive therapy for the management of bacterial corneal ulcer [103]. Steroids including sex hormones have been shown to inhibit collagen degradation by corneal fibroblasts in the collagen gel culture model [104,105]. Steroids thus attenuate both cell-mediated collagen degradation and the infiltration of mononuclear cells and consequent interaction between PMNs and corneal fibroblasts [17]. Controversy has long surrounded the clinical efficacy of steroids as an adjunctive therapy for bacterial keratitis [106,107]. Several clinical trials have been performed, but no concrete conclusions have been reached. A large-scale, well-controlled randomized clinical study (Steroids for Corneal Ulcers Trial) of adjunctive corticosteroid therapy for bacterial corneal ulcer showed improvement in visual acuity in patients other than those with *Nocardia* infection as well as no unfavorable reactions [108].

Triptolide is a compound produced by the plant species *Tripterygium wilfordii* Hook F and has been used in Chinese herbal medicine for the treatment of inflammatory diseases. Triptolide was also found to inhibit LPS-induced chemokine and adhesion molecule expression in, as well as IL-1-induced collagen degradation by, corneal fibroblasts [46,47,82], effects that may be mediated in part by attenuation of NF-κB signaling. Triptolide thus warrants further study as a potential therapeutic agent for corneal ulcer.

**Figure 3:**
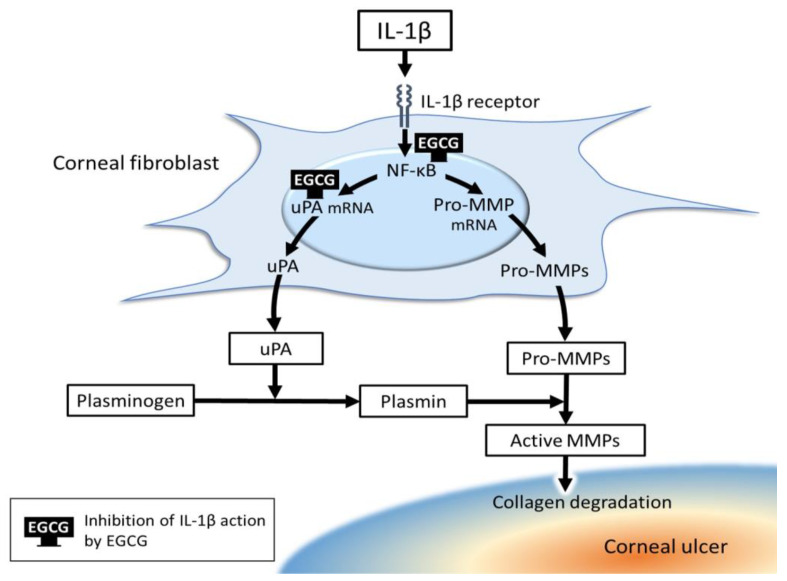
Possible mechanism for the inhibition by epigallocatechin-3-gallate (EGCG) of IL-1β-induced collagen degradation by corneal fibroblasts.

Another possible therapeutic target for corneal ulcer is the uPA-plasmin system. Epigallocatechin-3-gallate (EGCG) is the principal polyphenol in extracts of green tea and suppresses the IL-1β-induced up-regulation of uPA expression in and collagen degradation by corneal fibroblasts [109] (Figure 3). The target of EGCG for the suppression of collagen degradation is thus uPA rather than MMPs.

Finally, self-retaining cryopreserved amniotic membrane grafts are being pursued as another potential modality for the treatment of corneal ulcer associated with bacterial keratitis [110,111].

The future development of drugs for the treatment of corneal ulcer, which is characterized by the uncontrolled degradation of stromal collagen, will thus likely entail investigation of agents that target corneal fibroblasts themselves or individual components of the uPA-plasmin-MMP cascade that underlies collagen degradation by these cells. 

## 6. Conclusions

Bacterial infection of the cornea remains a serious medical problem. If the condition is diagnosed promptly and appropriate antibacterial treatment initiated, the clinical outcome is satisfactory in most cases. However, the emergence of drug-resistant bacteria poses a threat to this treatment approach.

The progression of bacterial keratitis to corneal ulcer also presents a therapeutic challenge. Once initiated, the degradation of corneal stromal collagen by corneal fibroblasts is able to proceed even after elimination of infecting bacteria. Furthermore, whereas the inflammatory reaction to invading bacteria is initially beneficial to the host, the persistence of this response can contribute to destruction of the normal structure and function of the cornea, with the resulting corneal opacity negatively affecting visual acuity and quality of life. Further insight into the pathobiology of infectious corneal ulcer is likely to inform the development of new treatment options. With regard to the study of collagen degradation, the culture system based on the embedding of corneal fibroblasts in a three-dimensional collagen gel remains an important experimental model that allows investigation of the interaction of various molecular factors as well as bacteria and inflammatory cells with corneal fibroblasts.

## Figures and Tables

**Table 1:** Phases of bacterial infection of the cornea.

Phase	Clinical Manifestation	Principal Players	Pathobiology	Treatment
Preceding event	Disruption of epithelial barrier and adhesion of bacteria	Bacteria	Trauma, CL wear, inflammation	Hygiene, proper CL handling, prophylactic administration of antimicrobials
Phase 1	Onset of keratitis	Bacteria (PAMPs), corneal fibroblasts, PMNs	Innate immune responses by corneal fibroblasts	Elimination of bacteria by antimicrobials
Phase 2	Onset of corneal ulceration	Bacteria	Collagen degradation by bacterial proteinases	Elimination of bacteria by antimicrobials. No drugs except steroids available to halt excessive stromal melting
		Corneal fibroblasts	Cell-mediated collagen degradation by uPA-plasmin-MMP cascade	
Phase 3	Prolonged corneal melting and perforation of the cornea	Corneal fibroblasts and PMNs	Vicious cycle between PMNs and corneal fibroblasts	Surgery including corneal transplantation

CL: contact lens; PAMP: pathogen-associated molecular pattern; PMN: polymorphonuclear neutrophil; uPA: urokinase-type plasminogen activator; MMP: matrix metalloproteinase.
